# Structural insights into the substrate-bound condensation domains of non-ribosomal peptide synthetase AmbB

**DOI:** 10.1038/s41598-022-09188-8

**Published:** 2022-03-30

**Authors:** Melissa-Jane Chu Yuan Kee, Sakshibeedu R. Bharath, Sheena Wee, Matthew W. Bowler, Jayantha Gunaratne, Shenquan Pan, Lianhui Zhang, Haiwei Song

**Affiliations:** 1 Institute of Molecular and Cell Biology, 61 Biopolis drive, Singapore, 138673 Singapore; 2 European Molecular Biology Laboratory, Grenoble Outstation, 71 Avenue des Martyrs, CS 90181, 38042 Grenoble, France; 3 Unit of Virus Host-Cell Interactions, Univ. Grenoble Alpes-EMBL-CNRS, 71 Avenue des Martyrs, CS 90181, 38042 Grenoble, France; 4 Department of Biological Sciences, National University of Singapore, Singapore, Singapore; 5 South China Agricultural University, 483 Wushan Rd., Tianhe Dist., Guangzhou, 510642 Guangdong Province China

**Keywords:** Microbiology, Structural biology

## Abstract

Non-ribosomal peptide synthetases (NRPS) are multi-modular/domain enzymes that catalyze the synthesis of bioactive peptides. A crucial step in the process is peptide elongation accomplished by the condensation (C) domain with the aid of a peptidyl carrier or thiolation (T) domain. Here, we examined condensation reaction carried out by NRPS AmbB involved in biosynthesis of l-2-amino-4-methoxy-trans-3-butenoic acid (AMB) in *P. aeruginosa*. We determined crystal structures of the truncated T–C bidomain of AmbB in three forms, the apo enzyme with disordered T domain, the holo form with serine linked phosphopantetheine (Ppant) and a holo form with substrate (l-alanine) loaded onto Ppant. The two holo forms feature the T domain in a substrate-donation conformation. Mutagenesis combined with functional assays identified residues essential for the attachment of Ppant, anchoring the Ppant-l-Ala in the donor catalytic channel and the role of the conserved His953 in condensation activity. Altogether, these results provide structural insights into the condensation reaction at the donor site with a substrate-bound C domain of AmbB and lay the foundation for understanding the molecular mechanism of condensation which is crucial for AMB synthesis.

## Introduction

*Pseudomonas aeruginosa* is a ubiquitous opportunistic Gram-negative pathogen that infects animals, plants and humans. It produces several virulence factors and secondary metabolites that contribute to its pathogenicity and overcome competition^1^. Among these secondary metabolites, an oxyvinylglycine, l-2-amino-methoxy-trans-3-butenoic acid (AMB), interferes with amino acid metabolism by inhibiting a wide array of pyridoxal phosphate-dependent enzymes^2^. In addition, AMB has been shown to prevent the growth of *E. coli* K12, *Bacillus *sp. and the plant pathogen *Erwinia amylovora*^3–5^.

AMB is synthesized in *P. aeruginosa* PAO1 by a five-gene cluster, *ambABCDE* that encodes two non-ribosomal peptide synthetases (NRPS), AmbB and AmbE; two iron containing (II)/α-ketoglutarate-dependent oxygenases, AmbC and AmbD; and a putative transporter, AmbA^6^ (Supplementary Fig. S1). AmbB and AmbE are large multi-domain enzymes made up of three or more conserved domains, each having a defined function in the biosynthesis of AMB (Supplementary Fig. S1). In NRPS, three essential domains are required for peptide assembly namely adenylation (A), peptidyl carrier or thiolation (T) and condensation (C) domain. The A domain is responsible for the specificity and activation of an amino acid using ATP as co-substrate, and the subsequent transfer of the activated monomer to the phosphopantetheine (Ppant) arm of a T domain. The tethered Ppant, which spans 20 Å, plays a central part in transporting covalently attached substrates to domains in the NRPS assembly line. Peptide elongation is achieved by the C domain, through amide bond formation between substrates bound to an upstream and a downstream T domain. Some NRPS incorporate modifications in the growing peptide through tailoring enzymes resulting in structural and functional diversity of non-ribosomal peptides^7^. In most bacteria, a thioesterase (TE) domain finally cleaves the final product from the NRPS, although in some cases, this step can be catalyzed by a reductase (R) domain^8,9^.

During AMB biosynthesis, the A domain of AmbB and AmbE activates l-Ala and l-Glu, respectively^10^. AmbB loads l-Ala on its own T domain and the T2 domain of AmbE, whereas the A domain of AmbE aminoacylates l-Glu on its T1 domain. Peptide elongation between l-Ala on AmbB and l-Glu on T1 of AmbE by the C domain of AmbB would then proceed. The resulting l-Ala-l-Glu would undergo condensation with l-Ala on T2 domain of AmbE forming the tripeptide l-Ala-l-Glu-l-Ala. Subsequently L-Glu would be modified by AmbC, AmbD and C domain of AmbE to give rise to l-Ala-AMB-l-Ala^10^. In a more recent study using deuterium labeling, it was shown that l-Glu gets sequentially hydroxylated at the C_3_ and C_4_ by AmbC and AmbD respectively, followed by methylation by MT domain of AmbE^11^. These modifications occur while l-Glu is still tethered to the T1 domain of AmbE prior to condensation with l-Ala from AmbB. It was also suggested that the resulting intermediate would undergo further modifications including dehydration (probably by the C domain of AmbE) and decarboxylation to generate the methoxy enol ether moiety^11^. The C domain of AmbE is atypical as phylogenetic analyses do not group this enzyme with other C domains (^L^C_L_, ^D^C_L_, starter, cyclization (C), epimerization (E), dual C/E)^10,11^. Finally, hydrolysis by thioesterase domain of AmbE would yield the end-product, Ala-AMB, instead of the tripeptide l-Ala-AMB-l-Ala that was formerly proposed^10,11^. Although these studies provide detailed information on AMB biosynthesis pathway, open questions remain, such as the precise role of the non-canonical C domain of AmbE, how are AmbC and AmbD recruited, and what enzyme is involved in processing the AMB precursor.

Many studies have focused on elucidating the function of individual domain of NRPS and domain–domain interactions. Structural data, combined with extensive biochemical and mutagenesis studies have provided some insights into the catalytic mechanism of condensation in NRPS^12–20^. The C domain, which has binding sites for one donor and one acceptor T domain, displays specificity towards both donor and acceptor substrates^13,21–23^. To date, existing structures of C domain include excised C domain, bidomains of C domain with either acceptor or/and donor T domain, or as part of an entire module of NRPS^14,18–20,24–27^. This gate-keeping mechanism, along with the unidirectional condensation reaction ensure correct elongation of product^28^. However, only few of the characterized structures of C domain contain Ppant tethered substrate in the donor or acceptor catalytic channel^22,26,27^.

In this work, we report the crystal structures of T–C bidomain of AmbB (AmbB T–C) in apo form, AmbB T–C with bound Ppant and AmbB T–C in complex with l-Ala loaded Ppant. Cell based bioassays and in vitro condensation assays coupled with mutagenesis, reinforce the indispensable role of active site histidine in the conserved HHxxxD motif of the C domain of AmbB. This work provides structural insights into a donor substrate-trapped state of a C domain, relevant for condensation reaction in NRPS.

## Results

### Overall structure description

**Figure 1:**
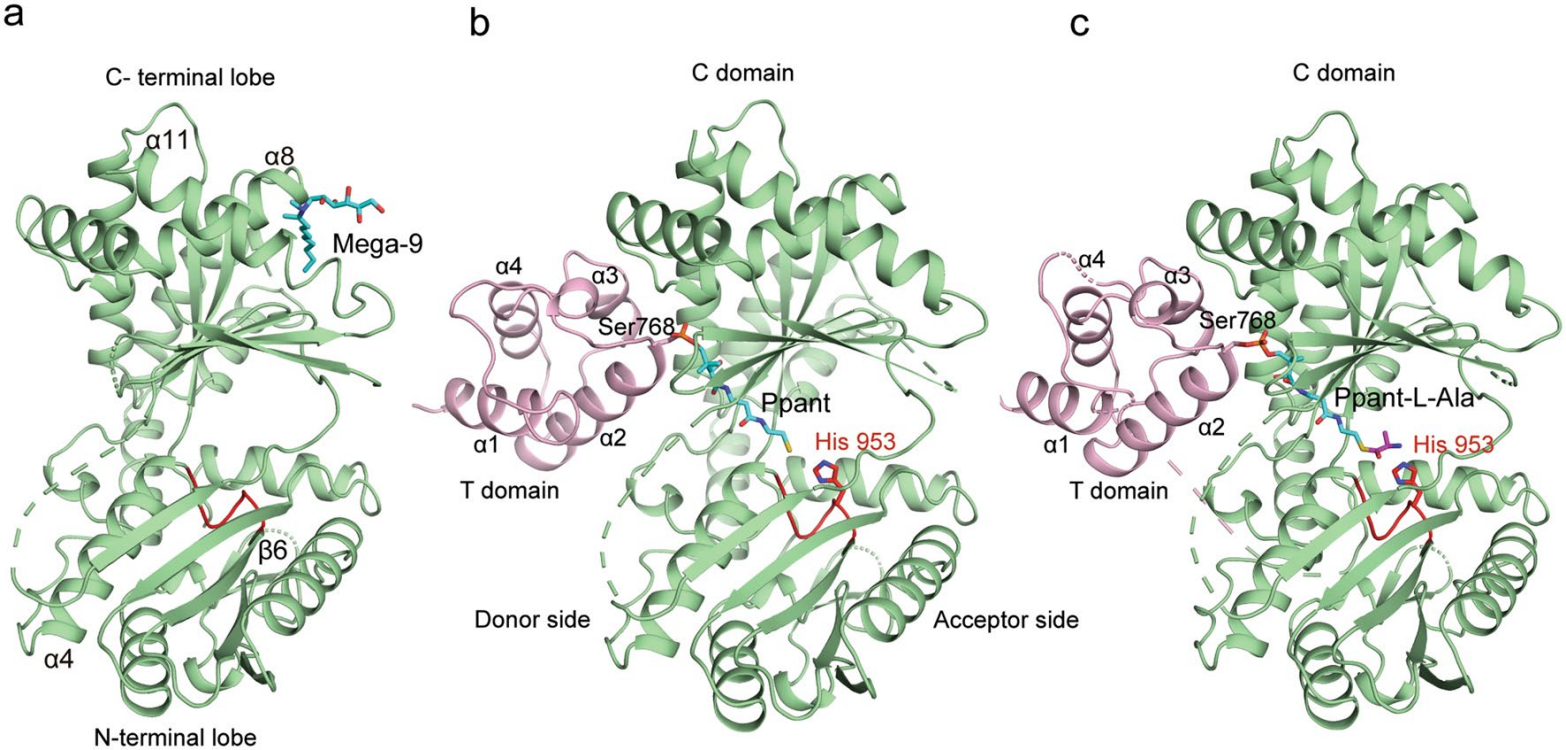
Cartoon representation of apo and holo AmbB T-C structures. (**a**) Structure of C domain of apo AmbB T–C with detergent MEGA-9 (cyan stick) bound to the C-terminal lobe, between helices α8 and α11 (**b**) Structure of holo AmbB T–C with Ppant (cyan stick) covalently attached to Ser768 situated at start of helix α2 of T domain. The C domain and T domain are colored green and pink, respectively. (**c**) Structure of holo Amb T–C with l-Ala (magenta) loaded to Ppant. Both holo AmbB T–C structures show that the T domain binds to the donor side of the C domain. The Ppant arm extends in the solvent channel towards the catalytically active His953 (red stick). The conserved HHxxxD motif is highlighted in red in all three panels. The disordered regions in the structure are represented as dashed lines.

Crystal structure of the apo form of AmbB T-C (residues 727–1249) was determined at a resolution of 2.4 Å (Supplementary Table S1). There is one molecule per asymmetric unit. The structure revealed a typical V-shaped condensation domain consisting of two subdomains, namely an N-terminal lobe (Gly824–Ser997) and a C-terminal lobe (Gly998–Ala1233) subdomains (Fig. 1a). The active site of the C domain is at the central groove formed by the two subdomains and has a conserved HHxxxD motif in a loop between strand β6 and helix α4 (Fig. 1a; Supplementary Fig. S2). There was no visible electron density for the T domain. This is likely due to inherent flexibility of the T domain as well as the absence of the cofactor, phosphopantetheine (Ppant). Residues constituting the loop region in the C domain, Gly881–Gly884, Lys1016–Arg1030, Asn1165–Asp1172 and Pro1198–Glu1200 are disordered and not built into the structure. Structural comparisons of the C domain of AmbB with other canonical bacterial C/E structures (CDA-C1, PDB 4JN3; VibH, PDB 1L5A; Srf A–C, PDB 2VSQ; TycC, PDB 2JGP; AB3403, PDB 4ZXH; GsrA, PDB 5ISW) revealed structural similarity among the condensation domains (RMSD in ranges of 2.2–2.8 Å) with closest similarity to Tyc-C6^24^ from *Bacillus brevis* for 351 α-C atoms. A detergent molecule from the crystallization condition, MEGA-9 is bound to the peripheral side of the C terminal subdomain between helix α8 and helix α11 (Fig. 1a). Such a binding mode far away from the active site is likely to be a crystallization artefact with no functional role. Similar feature is observed in the crystal structure of AB3403 wherein, the detergent 1,2-dimyristoyl-SN-glycero-3-phosphocholine is bound to the N-terminal lobe of the C domain^20^.

In order to understand the role of T domain and the Ppant arm in the condensation reaction catalyzed by the C domain of AmbB, we determined the crystal structures of holo AmbB T–C and AmbB T–C with l-Ala tethered to the Ppant arm at resolutions of 2.2 Å and 2.5 Å, respectively (Fig. 1b, c). The Ppant transferase, Sfp enzyme, was used to convert the apo form of AmbB T–C into its holo state. Mass spectrometry analysis of the two samples showed an increase mass of 318.1478 corresponding to Ppant-alkylated (Ppant-Alk) (Supplementary Fig. S3a, b), confirming the attachment of Ppant to Ser768 of T domain of AmbB. To obtain AmbB T–C with l-Ala, apo full-length AmbB, which activates l-Ala (Supplementary Fig. S4a), was used to trans-aminoacylate l-Ala onto the Ppant of the T domain of AmbB T–C. MS analysis showed a mass shift (+71.04) of the alkylated Ppant arm as compared to holo AmbB T–C, which corresponds to the molecular weight of l-Ala (Supplementary Fig. S3b, c).

Each of the two holo crystal structures have four T-C didomains in their asymmetric units. Helix 11 from the C domain which is partially disordered in the apo structure, is well defined in both the holo structures. However, the residues Asp813–Ala822 constituting the flexible linker region connecting the T and C domain are disordered and not observed in the electron density maps. Superposition of the two holo structures onto the apo structure shows that the C domains among the three structures are almost identical with RMSD values of 0.54–0.55 Å (holo AmbB T–C on apo AmbB: 327 α-C atoms; holo-AmbB T–C with l-Ala on apo AmbB: 317 α-C atoms). Likewise, the T domains between the two holo structures are very similar (RMSD: 0.26 Å for 58 α-C atoms) (Supplementary Fig. S5). The T domain occupies the donor binding site of the C domain, which supports its role as an aminoacyl donor for C domain of AmbB during the first elongation step of AMB biosynthesis. T domain is a four-helix bundle (Ser736–Gly812) that adopts an A/H configuration previously observed in apo and holo T domains of NRPS^29^ (Fig. 1b, c). The Ppant arm is covalently tethered to the invariant Ser768 in the conserved GGXS motif (Supplementary Fig. S6), situated at the start of helix α2 of the T domain. The orientation of the T domain, places Ser768 at the donor entry side of the C domain of AmbB, with the Ppant arm extending into the donor pocket towards the active site.

**Figure 2:**
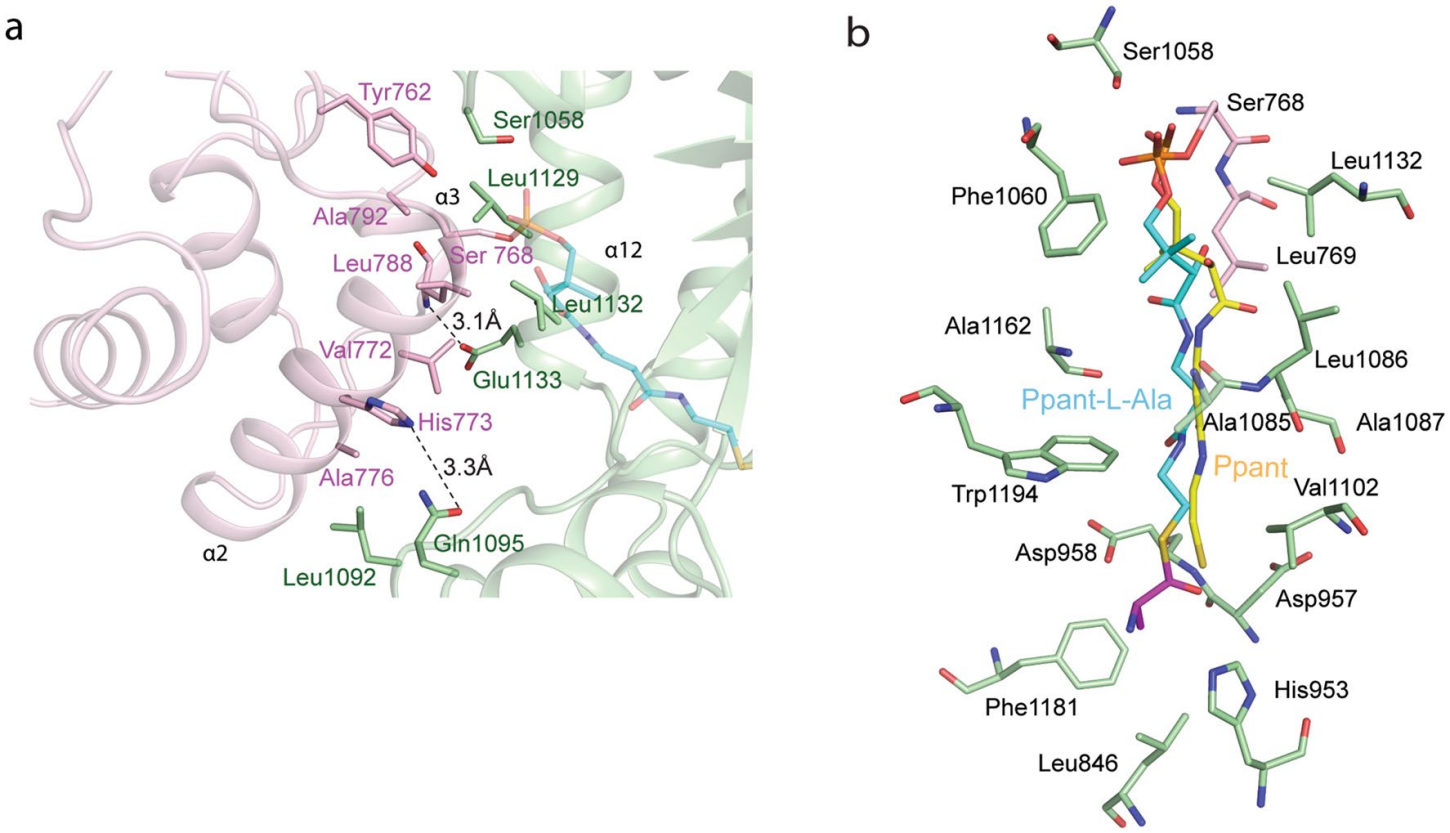
T–C interface and interaction of Ppant arm (with and without l-Ala) in C domain of AmbB. (**a**) Interaction of T–C domain in holo AmbB T–C (**b**) Interaction of Ppant arm (with and without l-Ala) in C domain of AmbB. The figure depicts superimposition of Ppant (yellow) and Ppant-l-Ala (cyan) (Chain B) in the donor pocket of C domain of AmbB. For simplicity, the residues involved in the interactions are shown from AmbB T–C tethered to l-Ala only.

As shown in Fig. 2a, besides the conserved Ser768 in T domain that anchors the Ppant arm in the C domain, T domain contacts C domain mainly via helices α2 and α3. The interactions at the T–C interface are predominantly Van der Waals contacts in nature with a buried surface area of 700 Å^2^. The Van der Waals interactions include residues Tyr762, Leu788, Ala792, Val772, and Ala776 from T domain and Ser1058, Leu1129, Leu1132 and Glu1133 and Leu1092 from C domain. The T–C interactions are further strengthened by a two hydrogen bonds. His773 from helix α2 is hydrogen bonded to Gln1095 situated on the η5 loop of the C domain. The amino group of Leu788 from the T domain forms a hydrogen bond with the carboxyl group of Glu1133 from helix α9 of the C domain.

### Interaction of Ppant arm (with and without L-Ala) in C domain of AmbB

Among the four protomers in the structures of holo AmbB T-C tethered with Ppant, only protomer D shows well defined electron density for Ppant but the ligand is in an unproductive configuration, where the thiol moiety does not point towards the putative catalytic His953. As shown in Fig. 2b, Ppant resides in the donor pocket that leads to the catalytic groove of the C domain. At the entrance of the pocket, one of the two phosphate oxygen atoms of Ppant is hydrogen bonded to Ser1058 from the C domain. Leu769 and Phe1060 form a hydrophobic pocket around the gem dimethyl moiety of Ppant. The walls of the pocket lining the Ppant are constituted of Ala1085, Leu1086, Ala1087, Ala 1162 and Tryp 1194. The terminal thiol end of the Ppant is surrounded by Val1102, two negatively charged residues namely Asp957 and the highly conserved Asp958, as well as the catalytic His953. The imidazole ring of His953 projects directly towards the donor channel, as observed in all structurally characterised C/E domains^14,18,19,24–26,30^.

In the structure of holo AmbB T–C tethered with Ppant-l-Ala, chains B and D exhibit relatively well-defined electron density for Ppant-l-Ala while chains A and C show fragmented density for Ppant-l-Ala (Supplementary Fig. S7). The interactions of Ppant moiety with C domain are essentially identical to those observed in the structure of holo AmbB T–C tethered with Ppant (Supplementary Fig. S8). The thiol moiety of Ppant is covalently attached to the carboxyl group of l-Ala via a thioester bond. The positioning of the Ppant places l-Ala in close vicinity to the active site, with the reactive carbonyl group facing the donor side of the C domain. l-Ala is surrounded by two residues namely Phe1181 and Leu846 (Fig. 2b).

### Structural comparison with T–C bidomain of LgrA

**Figure 3:**
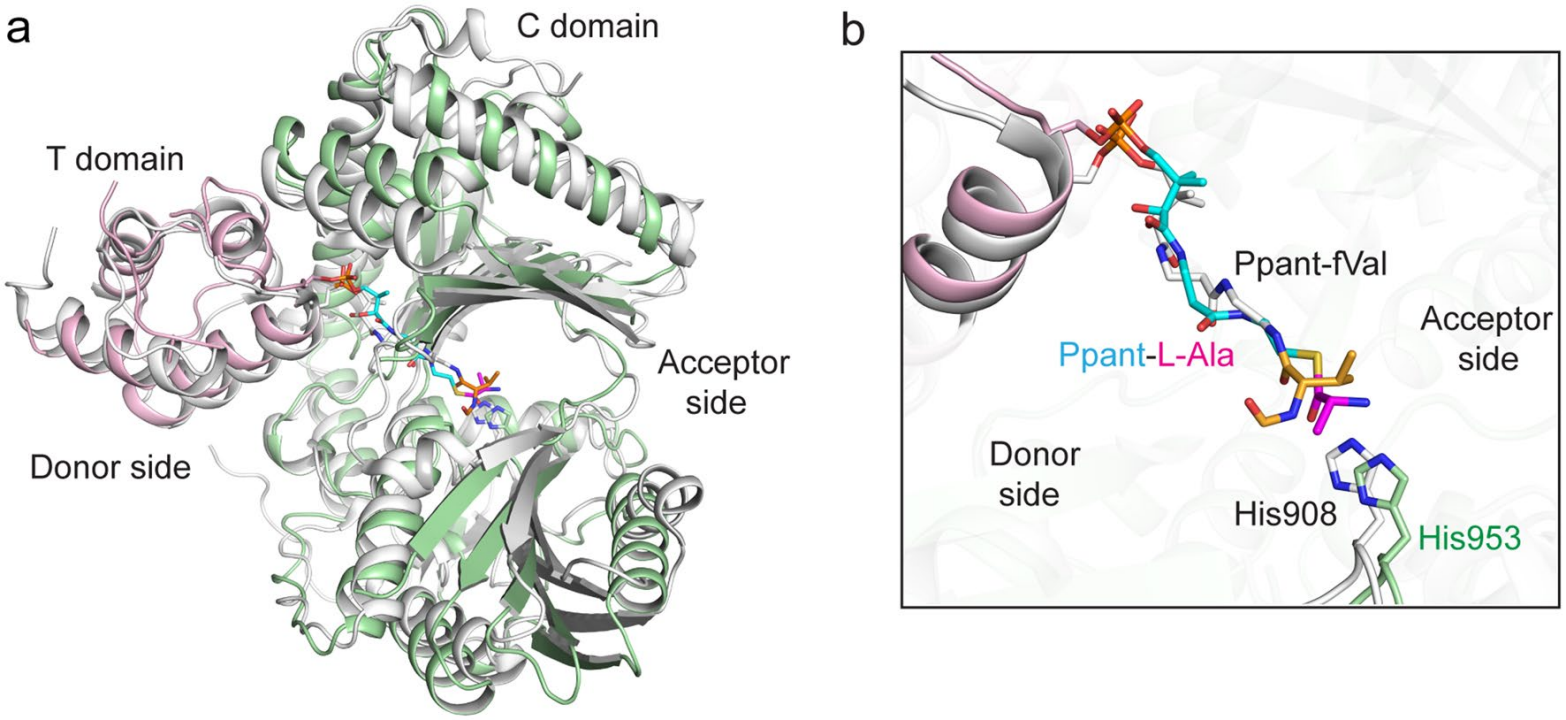
Superimposed structures of T–C bidomains of AmbB and LgrA. (**a**) In both structures, the T domain and C domain adopt similar orientation (RMSD value of 2.60 Å for 345 Cα). The T domain binds to the donor side of the C domain in both proteins. The F–A domains of LgrA is not shown for clarity. The T domain of AmbB is coloured pink and the C domain, green, whereas LgrA is coloured gray. (**b**) Close-up view of Ppant-L-Ala (yellow and magenta sticks) and Ppant-fVal (gray) in the C domain of AmbB (chain B) and LgrA, respectively. In both structures, Ppant-l-Ala and Ppant-fVal point towards the putative catalytic residues, His953 (AmbB) and His908 (LgrA).

Recently, the structure containing the full core (F1A1T1C2A2T2) of a dimodular NRPS LgrA from gramicidin synthetase was reported^26^. Superimposition of holo AmbB T-C with L-Ala and the T-C bidomain of LgrA, gave an RMSD value of 2.60 Å for 345 Cα, suggesting that AmbB T-C and T-C bidomain of LgrA adopt similar conformations (Fig. 3a). In the superimposed structure, the N and C subdomains of AmbB are seen in a more ‘open’ state as compared to LgrA. This can be accounted to the varying ‘openness’ of the protein as described among C domains of NRPS^18^. In the course of structure determination, formyl-valine (fVal)-CoA was chemically synthesised and subsequently covalently tethered to the T1 domain of LgrA in vitro. The structure showed that delivery of the substrate as well as simultaneous binding of donor T1 and acceptor T2 domains have no influence on the conformation of the C2 domain^26^. Ppant-l-Ala of AmbB follows the same trajectory in the donor side as Ppant-fVal, placing the substrate l-Ala and fVal, at similar distances to the catalytic His953 and His908, respectively (Fig. 3b). However, the orientations of l-Ala and fVal are different. In holo AmbB T–C with l-Ala (chain B), the side chain of l-Ala is oriented towards the hydrophobic residues Leu846 and Phe1181 while the free amino group is in close proximity to His953, poised for condensation reaction (Fig. 3b.). In LgrA, fVal needs to undergo a small rotation so that the reactive carbonyl carbon faces the acceptor side^26^.

### In vivo analysis of AMB production in *P. aeruginosa* mutants

**Figure 4:**
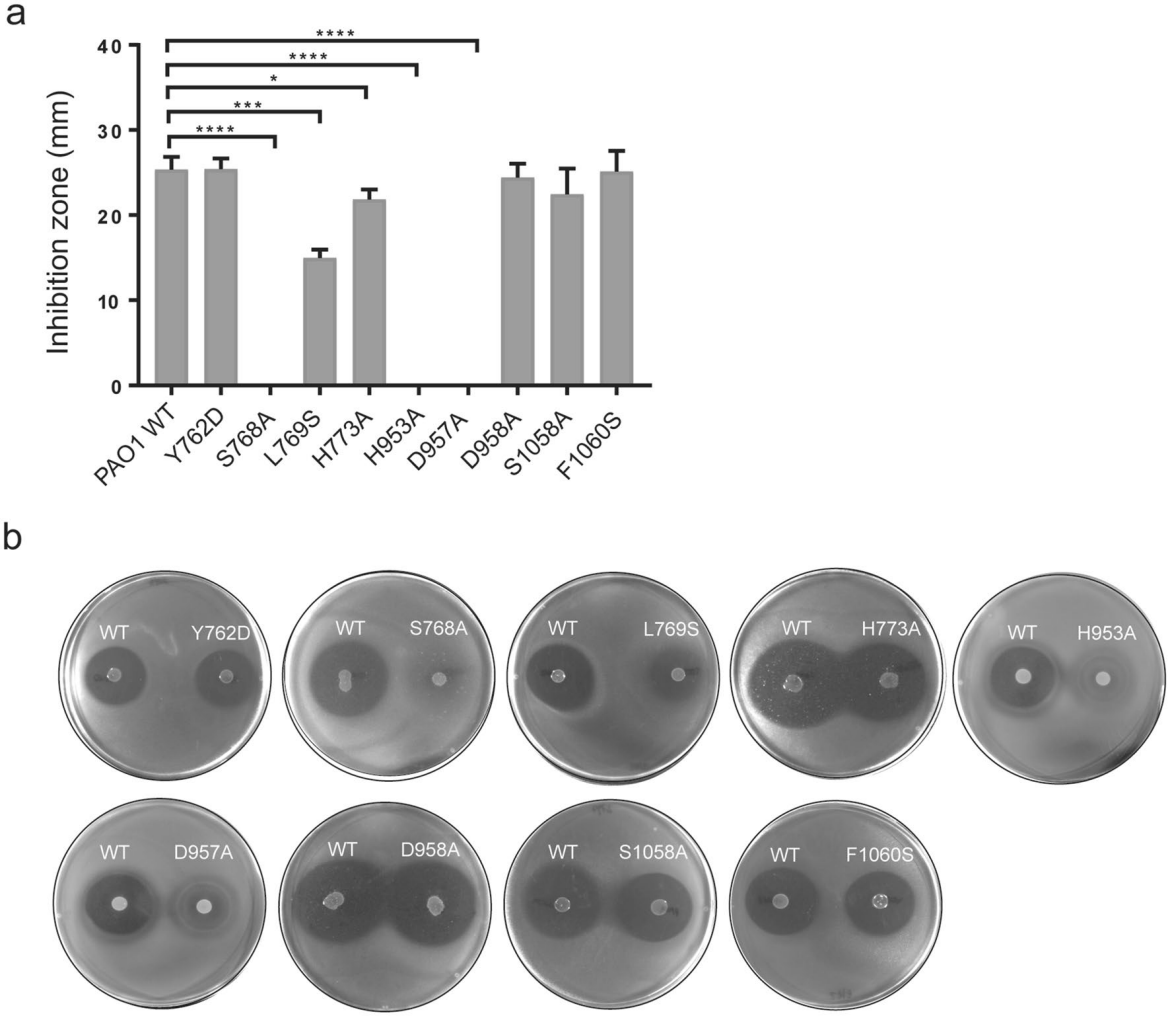
Plate inhibition assay for PAO1 WT and mutants against *E. coli* K12. (**a**) The diameter of zones of inhibition for PAO1 WT and mutants were measured using Image J software. Values are mean ± S.D (n = 3 independent samples). Any difference was considered significant at *P* value < 0.05 using *t*-test. *P < 0.0351; ***P < 0.006; ****P < 0.001. (**b**) In vivo AMB production results in zone of clearance around PAO1 WT or mutants due to growth inhibition of *E. coli* K12. PAO1 strains with Ser768A, His953A and Asp957A mutations showed no production of AMB, whereas PAO1 with Leu769S mutation exhibits significantly reduced AMB biosynthesis as compared to WT. AMB production was slightly significantly decreased for the mutant His773A. Tyr762D, Asp 958A, Ser1058A and Phe1060S showed no significant difference in AMB production.

Based on the residues involved in the interaction with Ppant-l-Ala (Fig. 2b), we constructed single site-specific mutations namely Ser768A, Leu769S, His953A, Asp957A, Asp958A Ser1058A and Phe1060S in the chromosome of *P. aeruginosa* PAO1. Subsequently, we assessed the mutants’ ability to produce the non-ribosomal peptide AMB in vivo using a plate bioassay based on the growth inhibition of *E. coli* K12^6^. *P. aeruginosa* wild type was able to produce a clear zone of clearance (25.33 mm ± 0.88) surrounding the spot inoculation whereas the mutant Ser768A did not display any zone of inhibition (Fig. 4a, b). Mutating the conserved Ser768, which is the attachment site for the Ppant arm on the T domain of AmbB, resulted in *P. aeruginosa* mutant being unable to produce AMB. Similarly, mutation of the second His953 and the conserved Asp957 in the conserved HHxxxD motif of the C domain resulted in abolition of AMB synthesis. The diameter of zone of inhibition for the mutant Leu769S was almost halved (14.97 mm ± 0.56), implying significant impaired AMB biosynthesis pathway. The mutants Asp958A, Ser1058A and Phe1060S did not show any significant difference in inhibitory activity in comparison to wild type *P. aeruginosa*. We also mutated two other residues Tyr762 (between helix α1 and α2) and His773 (in helix α2) on the T domain to assess the interaction at the T–C bidomain interface. Mutating Tyr762 to aspartate did not cause any disruption in AMB biosynthesis. The mutant His773A displayed a slight, but significant decrease in inhibitory activity with mean inhibition zone of 21.87 mm ± 0.66.

### Radioassay to assess condensation activity of C domain of AmbB

**Figure 5:**
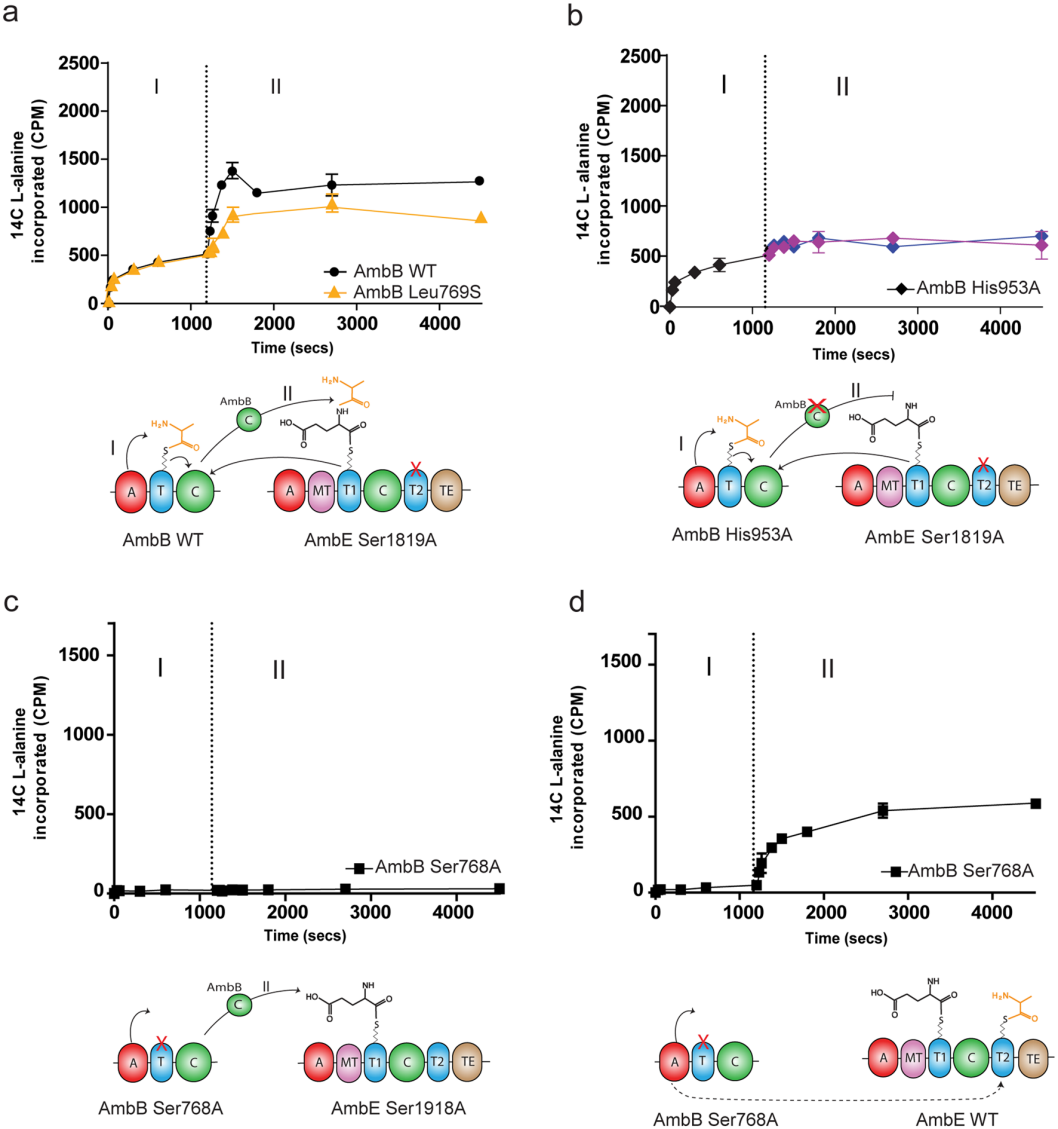
Time course of 14-C-l-Alanine precipitation radioassay for detection of l-Ala-l-Glu dipeptide synthesis. Product formation was initiated at 1200 s (represented by dotted line) by addition of an equimolar volume of pre-incubated AmbE S1819A with non-radiolabeled l-Glu (Stage II). (**a**) An almost doubling in 14-C-l-Alanine labeling was observed for AmbB WT which is due to dipeptide formation and rapid reloading of the T1 domain of AmbB WT. The condensation activity in mutant AmbB Leu769S is impaired as shown by the significantly decreased 14-C-l-Alanine labeling in Stage II. (**b**) Condensation activity is abolished in AmbB His953A as observed during stage II. The amount of 14-C-l-Alanine labeling for AmbB His953A with AmbE S1819A (blue line) was similar to that of AmbB His953A with buffer only (pink line). (**c**) Aminoacylation and dipeptide formation was abolished in AmbB Ser768. (**d**) Increasing l-[14C]-Ala labeling during Stage II was due to trans-aminoacylation of l-[14C]-Ala onto the T2 domain of AmbE WT by the A domain of AmbB Ser768A. The graph lines represent mean data ± SD obtained from three experiments. Any difference was considered significant at *P* value < 0.05 using *t*-test.

We next examined the condensation activity of full-length AmbB mutants in vitro, by using l-[14C]-Ala as indicator for formation of the dipeptide l-Ala-l-Glu. Aminoacylation of wild type or mutants AmbB was monitored for 20 min (Stage I), after which an equimolar amount of full-length AmbE Ser1819A that was priorly incubated with l-Glu, was added to initiate product formation (Stage II). Due to the mutation of the conserved Ser1819A on the T2 domain of AmbE, the dipeptide formed is not released from the protein and is stalled on the T1 domain of AmbE. Upon addition of AmbE Ser1819A to wild type AmbB, an increase equivalent to 867 CPM in l-[14C]-Ala labeling was detected. This almost doubling in l-[14C]-Ala labeling is attributed to the simultaneous translocation of l-[14C]-Ala to the T1 domain of AmbE as part of l-Ala-l-Glu dipeptide and rapid reloading of l-[14C]-Ala on the freed T domain of wild type AmbB (Fig. 5a). The synthesis of the l-Ala-l-Glu reached its peak at 5 min after addition of AmbE Ser1819A. As time proceeds, the amount of l-[14C]-Ala in the sample remains more or less constant since the amount of l-Ala-l-Glu and l-Ala on the T1 and T domain of AmbE and AmbB, respectively, reach saturation. As compared to wild type AmbB, the mutant Leu769S showed 50% decreased dipeptide synthesis (415 CPM) (Fig. 5a). For the mutant His953A, aminoacylation reaction was similar to wild type AmbB during Stage I. However, condensation activity seems to be completely abolished when AmbE Ser1819A was added, since l-[14C]-Ala labeling was comparable to that when AmbE Ser1819A was replaced with assay buffer only (Fig. 5b). The slight increase in l-[14C]-Ala labeling observed during Stage II can be explained by l-[14C]-Ala still being loaded on the T domain of AmbB His953A as saturation of the Ppant with l-[14C]-Ala was probably not achieved at 20 min. Consistent with the in vivo assay (Fig. 4), the mutant AmbB Ser768A which could not be modified with Ppant, lost its ability to aminoacylate l-[14C]-Ala on its own T domain during Stage I (Fig. 5c). Addition of AmbE Ser1819A did not result in the synthesis of dipeptide as no detectable labeling was observed (Fig. 5c). However, when AmbE Ser1819A was substituted with wild type AmbE in the reaction, a gradual increase in l-[14C]-Ala was observed during Stage II (Fig. 5d). Since A domain of AmbE does not show selectivity to l-Ala (Supplementary Fig. S4b), this increase is attributed to the loading of l-[14C]-Ala in trans on the T2 domain of wild type AmbE, as previously reported^10^.

### Mechanistic insights into the condensation reaction in AmbB

**Figure 6:**
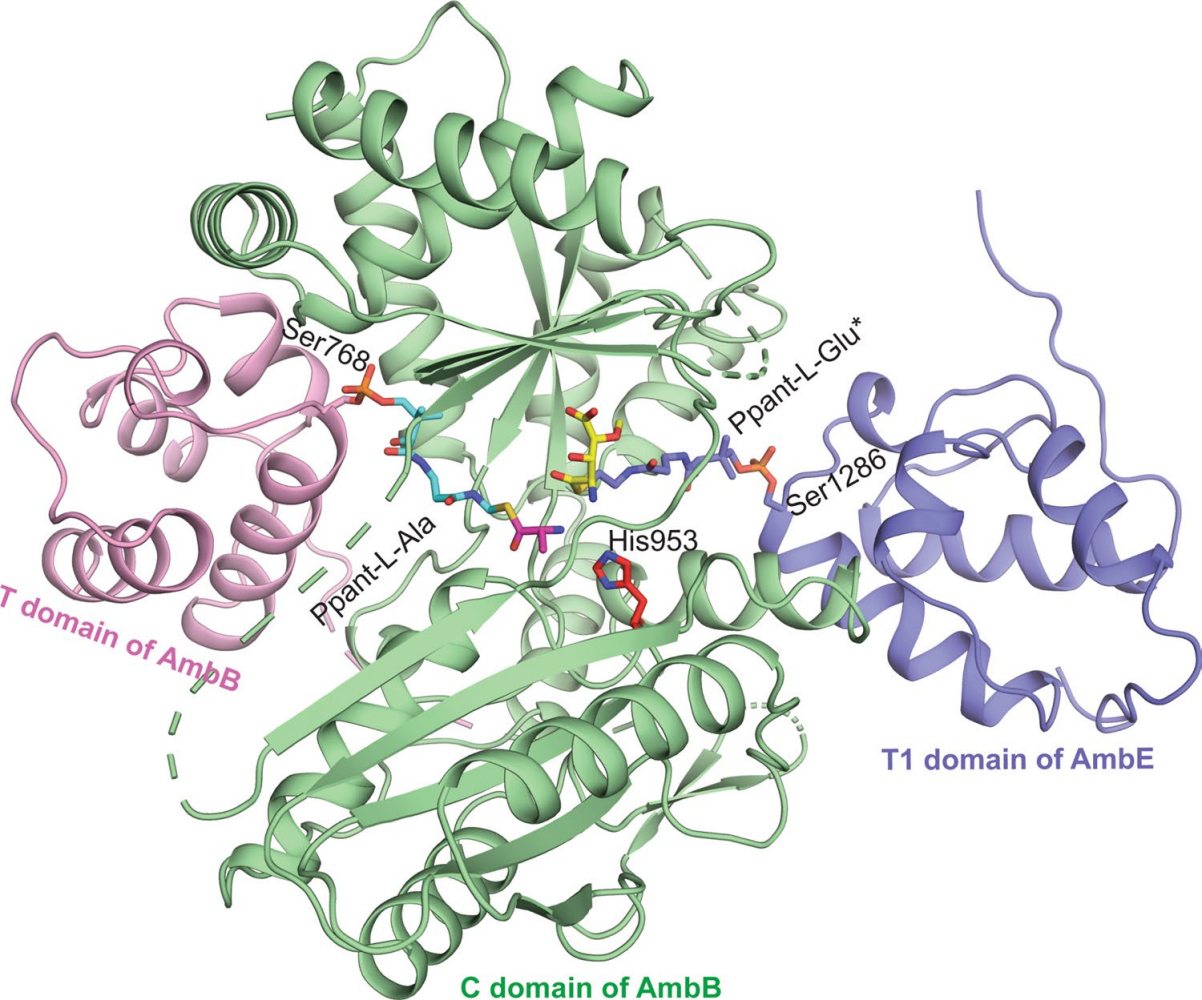
Model of the condensation complex formed by T–C bidomain of AmbB and T1 domain of AmbE. T domain of AmbB is on the donor side of its C domain while the T1 domain of AmbE is on the acceptor side. The amino acid substrates, l-Ala and l-Glu* (hydroxylated and O-methylated l-Glu) are attached to the Ppant arm of the respective T domains. The condensation complex brings the two amino acid substrates within the vicinity of the active site residue His953 and are poised for catalysis. The T1 domain of AmbE is shown in purple with bound Ppant-l-Glu* in purple stick. The coloring scheme of holo AmbB T–C with l-Ala tethered to the Ppant is as in Fig. 1c.

To gain a detailed insight into the mechanism of condensation reaction in AmbB, we modelled a hypothetical condensation complex using the C–T domains reported in the structure of holo-AB3403 (PDB: 4ZXH)^20^. In this model, the C domain of AmbB is sandwiched between the T domain of AmbB loaded with Ppant-l-Ala and T1 domain of AmbE loaded with Ppant-l-Glu* (hydroxylated and O-methylated l-Glu). Such an arrangement of the domains positions the two amino acid substrates in close proximity around the active site residue His953 so as to mimic the beginning step of the condensation reaction between the l-Ala and l-Glu* on the donor and acceptor sides of the C domain, respectively. The model places the ε N atom of the active His953 of AmbB at a reasonable distance of ~ 3.1 Å away from the amino group of the acceptor substrate, which would allow deprotonation of this α-amino group by His953 (Fig. 6). This distance is comparable to those observed between the equivalent catalytically active His157 and the acceptor substrate analogue in CDA-C1 (2.8 Å) (PDB: 5DU9)^22^, as well as between His2697 and the stabilised-Glycine acceptor substrate in PCP2-C3 (3.6 Å) (PDB:7KW0) of fuscachelin synthetase^27^. In this model, following deprotonation, the amino group of the acceptor substrate, which is positioned ~ 3.3 Å away from the Ppant-l-Ala thioester carbonyl, would then proceed with the nucleophile attack of the carbonyl group in order to release the l-Ala-l-Glu* dipeptide, tethered to the Ppant of the downstream acceptor T1 domain of AmbE.

## Discussion

The biosynthesis of NRPs requires the interplay of different domains and modules of NRPS. For peptide elongation, the C domain depends on the coordinated docking of two T domains for the delivery of substrates into the active site. The molecular mechanism of condensation is elusive due to limited number of structures with bound substrates. In this work, three crystal structures of T–C bidomain of AmbB were solved; apo form in which the T domain is disordered, and two holo forms with Ppant, or Ppant loaded with the native substrate, l-Ala, bound to the C domain. The importance of several residues in the donor solvent channel as well as in the active site of the C domain were assessed via site-specific mutagenesis, followed by in vitro and in vivo assays.

During the various stages of an NRPS synthesis cycle, the different domains form a dynamic catalytic platform while the mobile T domain handles the intermediates, by adopting different orientations relative to respective domains^20,25,26,31^. The presence of the T domain in the holo and AmbB T–C with Ppant-l-Ala bidomain structures showed that the Ppant arm locks the condensation-carrier domains in a stable conformation, and in turn stabilises the highly flexible carrier protein. It is also possible that AmbB may adopt a more compact fold in its holo state than apo form, as suggested by the shift in gel filtration peaks (Supplementary Fig. S9). Further biophysical analyses would be required to interrogate this possibility.

The C domains of NRPS exhibit different relative arrangement of the N-terminal lobe with respect to the C-terminal sub domain. This structural variability has previously led to suggest that the enzyme may adopt an ‘open’ and ‘closed’ state during various functional stages of NRPS biosynthesis^18^. Comparison to other homologue structures shows that the C domain of AmbB adopts a conformation quite similar to that in EntF from enterobactin synthetase (PDB: 5T3D) (Supplementary Fig. S10). The C domain of apo and holo AmbB T–C show a quasi-identical conformation suggesting that it does not undergo obvious conformation change upon Ppant binding, at least by its upstream T domain. In addition, the delivery of the substrate l-Ala, did not cause any discernible conformation change in the C domain.

Three structures of substrate-bound C domains of NRPS have been solved^22,26,27^. In addition to C domain of LgrA which features the synthesized donor CoA-fVal^26^, two other structures show mimics of acceptor substrate loaded Ppant in the C domain of CDA synthethase^22^, and stabilised-Glycine acceptor substrate in C domain of fuscachelin synthethase, respectively^27^. It is plausible that a mixture of Ppant and Ppant-l-Ala states may exist in the crystal structure of holo AmbB T–C with l-Ala due to the thioester linkage undergoing cleavage over the course of crystallisation. The Ppant arm which is intrinsically flexible as observed by the fragmented electron density of Ppant and Ppant-l-Ala in our structures, may also suggest that l-Ala may adopt different conformations in the active site of C domain of AmbB.

During AMB biosynthesis, the C domain of AmbB catalyses peptide bond formation between Ppant-l-Ala and Ppant-l-Glu* tethered on the donor T domain of AmbB and acceptor T1 domain of AmbE respectively^11^. Mutating the second Histidine (His953) to Ala in the consensus HHxxxDG motif of C domain was sufficient to abolish condensation activity of AmbB, thus reiterating its essential importance for catalytic activity. The dependence of C domain on the second His has also been demonstrated in EntF, TycB1 and SrfA-A, where a mutation to Ala/Val led to complete abolition of condensation activity^13,16,17,32^. However, little to moderate effect was reported for VibH and VibF^14,15^. Since the role of the second His seems not to be universally conserved in all C domains, it was recently proposed to be involved in positioning the acceptor substrate for nucleophilic attack, rather than acting as a general base in catalysis^22^.

In AmbB, an in vivo mutation of the well-conserved Asp957 has a detrimental effect on AMB biosynthesis as judged by the plate bioassay. Similarly, mutation of the equivalent Asp in TycB and Srf A–B was proven deleterious^12,17^. In EntF, an Asp to Met mutation not only abolished condensation activity, but also disrupted the global fold of the protein as confirmed by CD. This residue was assigned to be structurally important owing to its ionic interaction with a highly conserved Arg, rather than playing a direct role in catalysis^16^. In our study, the Asp957A mutant could not be purified as it underwent severe degradation, suggesting a crucial structural role in the C domain of AmbB. A 300-fold and 1500-fold decrease in catalysis was observed for VibH and CAT respectively, upon mutating the equivalent Asp residue to Ala^14,33^. In VibH, the decreased activity of the mutant is due to local active site perturbations^14^.

Functional analyses have also shown that the conserved Leu769 on the T domain of AmbB plays an important role in anchoring the Ppant in the donor site, as judged by its decreased AMB biosynthesis and condensation activity. This interaction, may in turn contribute to further stabilise the interaction between the T and C domain of AmbB, thus maintaining the T domain in a productive conformation during substrate delivery. Interestingly, the enterobactin synthethase EntF, bearing a mutation of the equivalent Leu (Leu1007A), could not be phosphopantetheinylated by EntD and Sfp, which the authors attributed to a defect in recognition of Leu1007 by Ppant transferase enzymes^34^. In our work, Leu769S mutant was able to auto-aminoacylate its own T domain with radiolabeled l-[14C]-Ala at the same rate as wild type AmbB, which showed that the decrease in dipeptide synthesis is probably due to its impaired interaction with the phosphopantetheine arm, rather than a defect in phosphopantetheinylation.

## Concluding remarks

This study provides structural insights into the donor substrate, l-Ala, in the active site of the C domain of AmbB. Using functional assays, we demonstrated that S768 is essential for the attachment of Ppant, while L769 is important for anchoring the Ppant-l-Ala in the donor site of the C domain of AmbB. Additionally, we showed that the second Histidine in the conserved HHxxxDG motif of C domain plays a crucial role in condensation activity. Further structural investigations of C domain with donor and/or acceptor substrates are needed in order to advance our knowledge on the mechanism of peptide synthesis in NRPS. This understanding is vital for successful and efficient NRPS bioengineering, which at present, still represents a challenge.

## Methods

### Bacterial strains, culture conditions and media supplements

*E. coli* and *P. aeruginosa* were routinely cultivated at 37 °C in lysogeny broth (LB) and agar. Purified stocks of bacteria strains were maintained at − 80 °C in LB containing 15% glycerol. When necessary, antibiotics were added to the media of *E. coli* strains at the following concentrations: ampicillin 100 μg ml^−1^, chloramphenicol 34 μg ml^−1^ and gentamycin 5 μg ml^−1^, For conjugation, *P. aeruginosa* was grown in LB medium at 42 °C overnight statically. Gentamycin at concentration of 60 μg ml^−1^ was used to select mero-diploids of *P. aeruginosa* following conjugation.

### Cloning of AmbB T–C, full-length AmbB and AmbE and their mutants

The gene encoding AmbB T–C bidomain was amplified using the High-Fidelity Q5 DNA polymerase (NEB) from genomic DNA of *P. aeruginosa* PAO1 using appropriate primers (Supplementary Table S2). The amplified fragment was digested with BamHI and XhoI and inserted into pGEX-6-P1 vector, which has a cleavable N-terminal GST tag. PCR-amplified fragment of full-length AmbB was digested with *Eco*RI and *Hind*III and ligated into pET28a vector, resulting in a plasmid (pET28SUMO-AmbB) which harbours a cleavable N-terminal SUMO tag. The amplified product of ambE was digested with *Nde*I and *Hind*III and cloned into pET21b vector with a C-terminal 6xHIS tag (pET21b-AmbE).

To generate full length AmbB mutants and AmbE S1819A, the plasmid pET28SUMO-AmbB and pET21b-AmbE was used respectively as template and amplified by inverse PCR using back-to-back primers. The amplified PCR product was incubated with DpnI, T4 PNK and T4 ligase for two hours at room temperature and 2 μl of the reaction mixture was transformed into chemically competent *E. coli* DH5α. Positive transformants were confirmed by sequencing and the plasmid transformed into *E. coli* BL21 Codon Plus for overexpression.

### Construction of *P. aeruginosa* mutants

The plasmid pET28SUMO-AmbB was used as template to amplify a 1000 bp-gene carrying respective single amino acid substitution (Supplementary Table S2). For ambB gene replacement in *P. aeruginosa*’s chromosome, the amplified 1000 bp gene fragment was digested from pET28aSUMO vector using *Eco*RI and *Hind*III restriction enzymes and ligated into the suicide vector pk18-Gm-mobsacB between the same sites. Using S17-1 λpir *E. coli* as donor cells, the suicide plasmid was mobilized from *E. coli* DH5α into *P. aeruginosa*.

Single homologous recombination event between ambB gene on the chromosome and the mutator fragment on pk18-Gm-mobsacB was confirmed by selection for gentamycin resistance. Excision of the vector resulting from the second recombination event was achieved on LB plates without sodium chloride (NaCl), supplemented with 10% sucrose. Single colonies were screened using primers upstream and downstream of *ambB* to confirm the right insertion of the homologous mutator fragment into the chromosome. Single amino acid substitution was confirmed by sequencing.

### Overexpression of AmbB T–C bidomain

Cultures were set up in two flasks each containing 2 l of LB and each flask was inoculated with 20 ml of an overnight culture of *E. coli* Codon Plus carrying the relevant plasmid. The cultures were grown at 37 °C until OD600 reached 0.5–0.7 and induced with a final concentration of 0.1 mM isopropyl-β-d-thiogalactopyranoside (IPTG), after which growth was continued for another 16 h at 18 °C.

### Overexpression of full length AmbB WT, AmbB mutants, AmbE WT and AmbE S1819A

2 L of LB were inoculated with an overnight culture of *E. coli* Codon Plus (AmbB WT and AmbB mutants) and *E. coli* pLysS (AmbE WT and AmbE S1819A). At OD600 of 0.5–0.7, IPTG at a final concentration of 0.5 mM was used for induction and the cultures were grown for another 4 h at 24 °C.

### Purification of AmbB T–C

Following heterologous expression, cultures were centrifuged at 4000 RCF at 4 °C for 30 min. Supernatant was discarded and pellet was resuspended in 100 ml buffer A containing 50 mM Tris pH 8.0, 500 mM NaCl, 12% v/v glycerol and 2 mM dithiothreitol supplemented with 2 mM magnesium chloride (MgCl2), 500 μl lysozyme (10 mg ml^−1^), 1 mM phenylmethylsulfonyl fluroride (PMSF) and 2 mM benzamidine hydrochloride. The solution was incubated on ice for 30 min prior to sonication. A total of 5 min (03 s on; 15 s off) was used to lyse the resuspended pellet. The soluble and insoluble fractions were separated by centrifugation at 18,000 RCF at 4 °C for 30 min. The supernatant was filter sterilized using 0.22 μM filters and incubated with 5 ml of gluthatione sepharose beads for 30 min at 4 °C. The mixture was loaded onto a polypropylene column, after which the beads were washed with 50 ml buffer A to remove unbound contaminants. GST-tagged AmbB T–C protein was then eluted with 50 ml of 20 mM reduced glutathione prepared in buffer A. To cleave the N-terminal GST tag from AmbB T–C protein, the evaluate was incubated with precision protease in a ratio of 50:1 for 2 h at 4 °C. The mixture was passed through a desalting column and then bound again to GST beads to remove GST tag. The flow-through containing AmbB T–C protein was collected and was purified further on an anion exchange chromatography column (Mono Q 10/100, GE Healthcare) using 20 mM Tris pH 8.0, 50 mM NaCl and 20 mM Tris pH 8.0, 1 M NaCl as running and eluting buffer respectively. AmbB T–C protein was lastly purified on a Superdex 75 column (HiLoad 16/600, GE Healthcare) using buffer B consisting of 20 mM Tris pH 8.0, 150 mM NaCl and 2 mM DTT. Pure AmbB T–C was subsequently concentrated with Amicon Ultra centrifugal filter with molecular weight cut–off of 10,000. Concentration of AmbB T–C was determined based on the calculated extinction coefficient and the protein (55 mg ml^−1^) was flash frozen in liquid nitrogen and stored in aliquots at − 80 °C.

### Purification of full length AmbB WT, AmbB mutants, AmbE and AmbE S1819A

Buffer A containing no DTT was used for cell lysis for AmbB WT, AmbB mutants, AmbE and AmbE S1819A. Following centrifugation at 18,000 RCF for 30 min, the supernatant was filter-sterilized and incubated with 5 ml NiNTA beads for 1 h at 4 °C. The mixture was loaded onto a polypropylene column and the beads were washed with 50 ml buffer A containing 20 mM imidazole to remove contaminants. To elute the His-tagged proteins, 50 ml of buffer A containing 200 mM imidazole was used. Respective protein was then concentrated to 5 ml and applied to a Superdex 200 column (HiLoad 16/600 Prep Grade, GE Healthcare) that had been equilibrated with buffer B. After purification, fractions of pure proteins were concentrated with Amicon Ultra centrifugal filter with molecular weight cut-off of 30,000. Concentration of AmbB WT, AmbB mutants, AmbE and AmbE S1819A were determined based on the calculated extinction coefficient and respective protein was flash frozen in liquid nitrogen and stored in aliquots at − 80 °C.

### In vitro phosphopantetheinylation assay

The conversion of apo to holo form of proteins was done prior to size exclusion chromatography step. In a 500 μl reaction, concentrated protein was incubated with 5 mM coenzymeA, 1 μM sfp enzyme (NEB) and 10 mM MgCl2. The mixture was incubated at 28 °C for 2 h.

### Loading of l-Ala in trans onto T domain of AmbB T–C

Apo full length AmbB was used to load L-alanine onto the T domain of the holo truncated AmbB T–C. 100 μM of full length AmbB was mixed with 500 μM AmbB T–C in an assay buffer containing 20 mM Tris pH 8.0, 150 mM NaCl, 5 mM ATP, 5 mM l-Ala and 10 mM MgCl_2_. The reaction mixture was incubated at 28 °C for 3 h followed by overnight incubation at room temperature. The mixture was run on a mono Q column to separate full length AmbB from truncated AmbB T–C. The samples corresponding to AmbB T–C was pooled, concentrated and further purified on a Superdex 75 size exclusion chromatography column.

### Crystallization of Apo and holo AmbB T–C

Crystallization of apo AmbB T-C was reported previously^35^. For holo AmbB T-C, a total of 480 conditions were screened using commercially available kits (Hampton Reasearch and Qiagen). 0.2 μl of holo AmbB T–C protein at concentration of 10 mg ml^−1^ was mixed with 0.2 μl of precipitant solution and equilibrated against 56 μl of reservoir condition. After one day at 18 °C, crystals were observed in several conditions. Optimization of the initial hits using the hanging drop method led to single plate-like crystals that grew in a condition containing 0.1 M MES pH 6.6, 0.2 M ammonium nitrate, 15% PEG 3350 and 5% glycerol. A typical drop consisted of 2 μl protein with 2 μl precipitant against 120 μl of reservoir in 48-well crystallization plates (Hampton Research). Crystals were soaked briefly in a cryoprotectant solution made up of mother liquor supplemented with 25% (v/v) 2-methyl-2,4-pentanediol (MPD) and harvested with cryoloops (CrystalCap HT). Crystals were flash- frozen in liquid nitrogen and sent for X-ray analysis at the European Synchroton Radiation Facility in Grenoble, France. Crystals of holo AmbB T–C with l-alanine grew in the same original condition as AmbB T–C. The crystals were harvested in mother liquor containing 0.2 M ammonium nitrate, 15% PEG3350 and 25% MPD and flash frozen in liquid nitrogen.

### Data collection and structure determination

Crystals structure of apo T–C AmbB were determined using Set-Met labeled protein which diffracted to 2.40 Å, with space group P6_5_22, unit-cell parameters a = b = 87.8 Å, c = 286.3 Å, α = β = 90°, γ = 120°, and contained one molecule per asymmetric unit. A diffraction dataset was collected at SSRF (BL17U1) and was processed using CCP4^36^. The AmbB-T–C domain has only two methionine sites in 500 amino acids. We introduced three additional methionines in place of leucines and prepared cement crystals suitable for SAD phasing. These crystals diffracted poorly after initial 30–40 frames of data collection. So, we merged datasets from four different crystals to obtain initial SAD phases using HySS program of phenix suite^37^. Five sites were identified with a figure of merit of 0.2 and correlation of local RMS density of 0.68 suggesting a possible correct solution. Subsequent refinement of heavy atom positions, phasing and density modification using RESOLVE allowed identification of the correct handedness of the map. Initial model was built with 391 amino acids in nine different fragments corresponding to one chain of AmbB-T-C domain with a map-model correlation of 0.834.

Crystals of holo T-C AmbB was solved at a resolution of 2.2 Å, with space group C121, unit-cell parameters a = 198.6 Å, b = 71.4 Å and c = 172 Å, α = γ = 90°, β = 109. 9°, and contained four polypeptides per asymmetric unit. The structure was determined by molecular replacement method using Phaser, with the structure of C domain of apo AmbB T–C as search model. The T domain of AmbB was manually built based on available density map using COOT, and crystallographic refinement was performed using REFMAC. Crystals of holo T–C AmbB with Ppant-l-Ala diffracted to 2.5 Å, with the same space group and cell parameters as holo AmbB T–C. The structure was auto built using Phenix and using holo AmbB T-C as model. Statistics of the structure determination and refinement are summarized in Supplementary Table S1.

### Plate bioassay for in vivo detection of AMB production in *P. aeruginosa* strains

AMB production was assessed by the ability of *P. aeruginosa* strains to inhibit *E. coli* K12^6^. Wildtype and mutant *P. aeruginosa* strains were grown in GYP medium (1% v/v glycerol, 0.4% bacto peptone, 0.4% bacto yeast extract for 48 h. 5 μl of culture from the wild type and mutant was spotted on minimal medium E (MME) plates supplemented with 0.5% glucose. The plates were incubated at 37 °C for 16 h and then exposed to ultra violet (UV) light for 5 min. 4 ml of soft agar (0.5% w/v) inoculated with 0.4 ml of *E. coli* K12, grown overnight in MME medium supplemented with 0.5% glucose and 1 mM threonine, and brought to an OD600 of 0.1 with 0.9% NaCl, was overlaid on the MME plates. Plates were incubated at 37 °C for 24 h. The zones of clearance around *P. aeruginosa* spots were visually assessed and measured using Image J software. The assay was done in triplicates (n = 3 independent samples) for each mutant. Unpaired *t*-test in GraphPad Prism was used to analyze any significant difference in the diameter of zones of clearance between wild type and mutants. Data was considered significant at a *p* value of ≤ 0.05.

### Adenylation activity of AmbB and AmbE

A colorimetric detection of pyrophosphate was used as indicator to assess the substrate specificity for the A domain of AmbB and AmbE^38^. Briefly, 100 μg ml^−1^ of AmbB or AmbE was added to a tube containing 50 mM Tris pH 8.0, 5 mM ATP, 5 mM MgCl_2_, 32 mM hydroxylamine (pH 7.2), 2 mM l-Ala or l-Glu and the reaction mixture was topped up with water to 100 μl. The mixture was incubated at 30 °C for 60 min. 50 μl of the reaction was then transferred to an Eppendorf tube containing 500 μl of disodium molybdate (VI) dihydrate solution and incubated at room temperature for 3 min. 10 μl of *bis* (triphenylphosphoranylidene) ammonium chloride (BTPPACl) in acetonitrile was added, and the reaction mixture was further incubated for 5 min. The sample was centrifuged at 13,000 RPM for 10 min and the supernatant was discarded. The precipitant was dissolved in 100 μl of acetonitrile and mixed thoroughly. 10 μl of the solution was transferred to a 96-well flat bottom plate, to which 10 μl of ascorbic acid solution (0.44 g ascorbic acid, 2 mL 5 M HCL, 3 mL acetonitrile) was added. The mixture was pipetted a few times and absorbance of the well was read at 620 nm. For negative control, amino acid solution was substituted with water. The readings were compared to a standard curve, which was performed with sodium tetrasodium pyrophosphate using the same above described method.

### Radioassay for the detection of l-ala-l-glu dipeptide formation

To assay the in vitro condensation activity of wildtype and mutant full length AmbB, we used radiolabeled 14C-l-alanine to assess dipeptide formation. A mutant AmbE_S1819A was generated so as to abolish loading of l-alanine in trans by AmbB onto the T2 domain of AmbE, that could interfere with the 14C-l-ala-l-glu reading. 1 μM of AmbES1819A was pre-incubated with 5 mM non-radiolabeled l-glutamic acid, 5 mM ATP and 5 mM MgCl_2_ for 1 h at room temperature. The aminoacylation reaction of wildtype or mutant AmbB in assay buffer containing 5 mM ATP and 5 mM MgCl_2_ was started by adding 4 μM radiolabeled 14C l-alanine. At 20 min time point, product formation was initiated by mixing equal volumes of pre-incubated AmbE_S1819A with wildtype or mutant AmbB. At various time points, 25 μl aliquots were taken and quenched immediately with ice-cold 10% w/v TCA containing 100 μl of 100 mg ml^−1^ BSA. The samples were centrifuged at 12,000 RCF for 5 min and washed twice with 10% TCA. The final pellet was resuspended in 150 μl formic acid and added to 3 ml of LSC fluid (Ultima Gold). 14C-radioactivity in the samples were quantified by LSC.

### Liquid chromatography-mass spectrometry analysis

To confirm the covalent attachment of Ppant or Ppant-l-Ala on AmbB T–C, 60 μg of protein in 60 μl 20 mM Tris (pH 8.0) solution containing 150 mM sodium chloride and 2 mM DTT (Sigma-Aldrich) was reduced by the addition of 6 μl 55 mM DTT and incubation at room temperature for 30 min. This was followed by addition of 6 μl of 120 mM iodoacetamide (Sigma-Aldrich) and incubation at room temperature in the dark for 30 min. 6 μl of 0.2 μg μl^−1^ trypsin (Promega) was then added to the sample and incubated further at 37 °C overnight. The digested sample was treated with 100 μl 0.1% formic acid to stop trypsin digestion and the sample was cleaned up using stage tip^39^. The sample was stored in the stage tip at 4 °C until it is ready for mass spectrometry analysis. The sample eluted from the stage tip was vacuum-dried, reconstituted in 30 μl 0.1% formic acid and diluted to 0.5 μg μl^−1^ for liquid chromatography mass spectrometry analysis.

2 μg of peptides was analyzed on Thermo Easy nLC 1000 that was connected to Q Exactive Plus Mass Spectrometer (Thermo Scientific). The trap column used was C18 Acclaim PepMap 100 (5 μM, 100A, 100 μM I.D. × 2 cm) and the analytical column was PepMap RSLC C18 (2 μM, 100A, 75 μM I.D. × 50 cm). Mobile phase A was 2% acetonitrile/0.1% formic acid while mobile phase B was 95% acetonitrile/0.1% formic acid. The separation of the peptides was carried out with a flow rate of 200 nl/min at 50 °C using a 95 min gradient: 0–34% mobile phase B for the first 70 min, followed by a 10 min gradient ranging from 34 to 100% mobile phase B and maintained at 100% mobile phase B for 15 min. The mass spectrometer was set in the data dependent acquisition mode. Full scan MS spectra (*m/z* 310–2000) were acquired with a resolution of 7e5 at an AGC target of 3e6, and a maximum injection time of 10 ms. Top 20 most intense peptide ions in each MS scan were sequentially isolated to an ACG target value of 5e4 with resolution of 17,500 and fragmented using a normalized collision energy of 25 at MS2 level with fixed first mass at 110 and isolation window of 2.0. The mass spectrometry data was processed manually by performing “Extracted Ion Chromatogram” on the product ion and the MS/MS spectra was manually interpreted.

### Modeling the condensation complex of AmbB–AmbE

Our structure of TC bidomain of AmbB with Ppant-l-Ala represents one-half of the condensation complex. First, we modeled the T1 domain of AmbE based on the T–C domain reported in the structure of holo-AB3403 wherein the T domain is situated in the acceptor site of its C domain (PDB: 4ZXH)^20^. Next, we superposed the C domains of 4ZXH and AmbB-Ppant-l-Ala to obtain the condensation complex of AmbB–AmbE. As expected, the modeled T1 domain occupied a position on the acceptor side of the C domain of AmbB. l-Glu* was manually connected to the Ppant bound to the acceptor site of the C domain of AmbB and the whole condensation complex was subjected to rigid body and energy minimization refinement to optimize the interactions between the modeled T1 domain of AmbE and the C domain of AmbB. In the resultant condensation complex, both the acceptor and donor sites on the C domain of AmbB are occupied by Ppant-l-amino acid loaded T domain.

## Supplementary Information

Supplementary Information.

## Data Availability

Atomic coordinates and related structure factors for Apo AmbB T–C, Holo AmbB T–C and Holo AmbB T–C with l-Ala have been deposited in the Protein Data Bank with codes 7X0E, 7X0F, 7X17, respectively.
