# Supplementary material for: Structural insights into the substrate-bound condensation domains of non-ribosomal peptide synthetase AmbB
Source: Sci Rep. 2022 Mar 30;12:5353. doi: 10.1038/s41598-022-09188-8 (PMC8968710; doi:10.1038/s41598-022-09188-8)
Supplement: Supplementary file 1 — Supplementary Information. [file 41598_2022_9188_MOESM1_ESM.pdf]

SUPPLEMENTARY INFORMATION

Structural insights into the substrate-bound condensation domains of non-ribosomal peptide synthetase AmbB

Melissa-Jane Chu Yuan Kee[1#], Sakshibeedu R Bharath[1#], Sheena Wee[1], Matthew W Bowler[2,3], Jayantha Gunaratne[1], Shenquan Pan[4], Lianhui Zhang[5] and Haiwei Song[1,*]

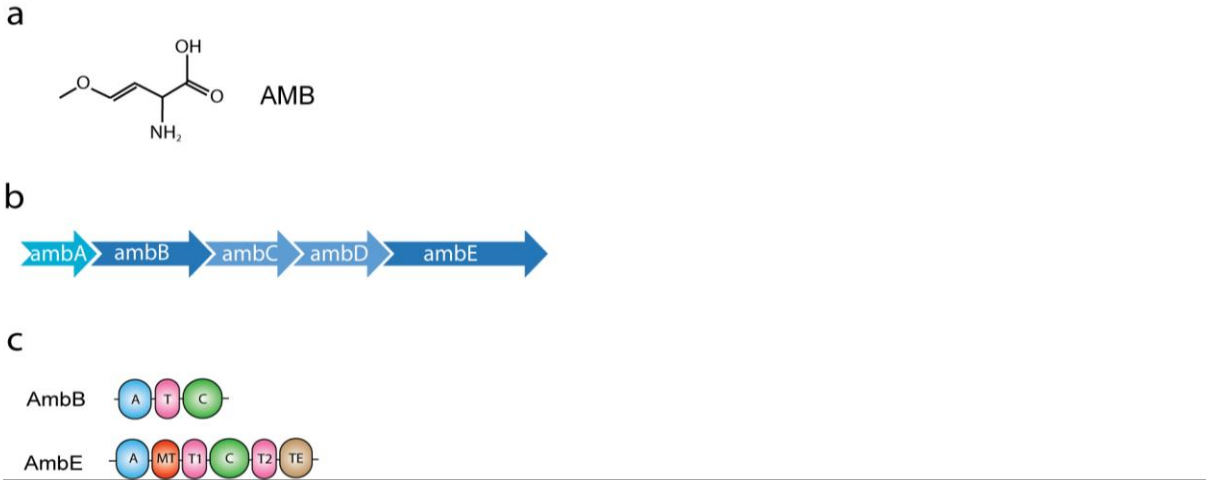

Supplementary Figure. 1 Schematic representation of *amb* operon and NRPS AmbB and AmbE in *P. aeruginosa* PAO1. (a) Structure of non-ribosomal peptide AMB **(b)** *amb* biosynthetic gene cluster encoding one LysE transporter (ambA), two Fe(II)/α–ketoglutarate monooxygenases (AmbC and AmbD) and two NRPS (AmbB and AmbE) **(c)** Domain architecture of AmbB and AmbE NRPS modules.

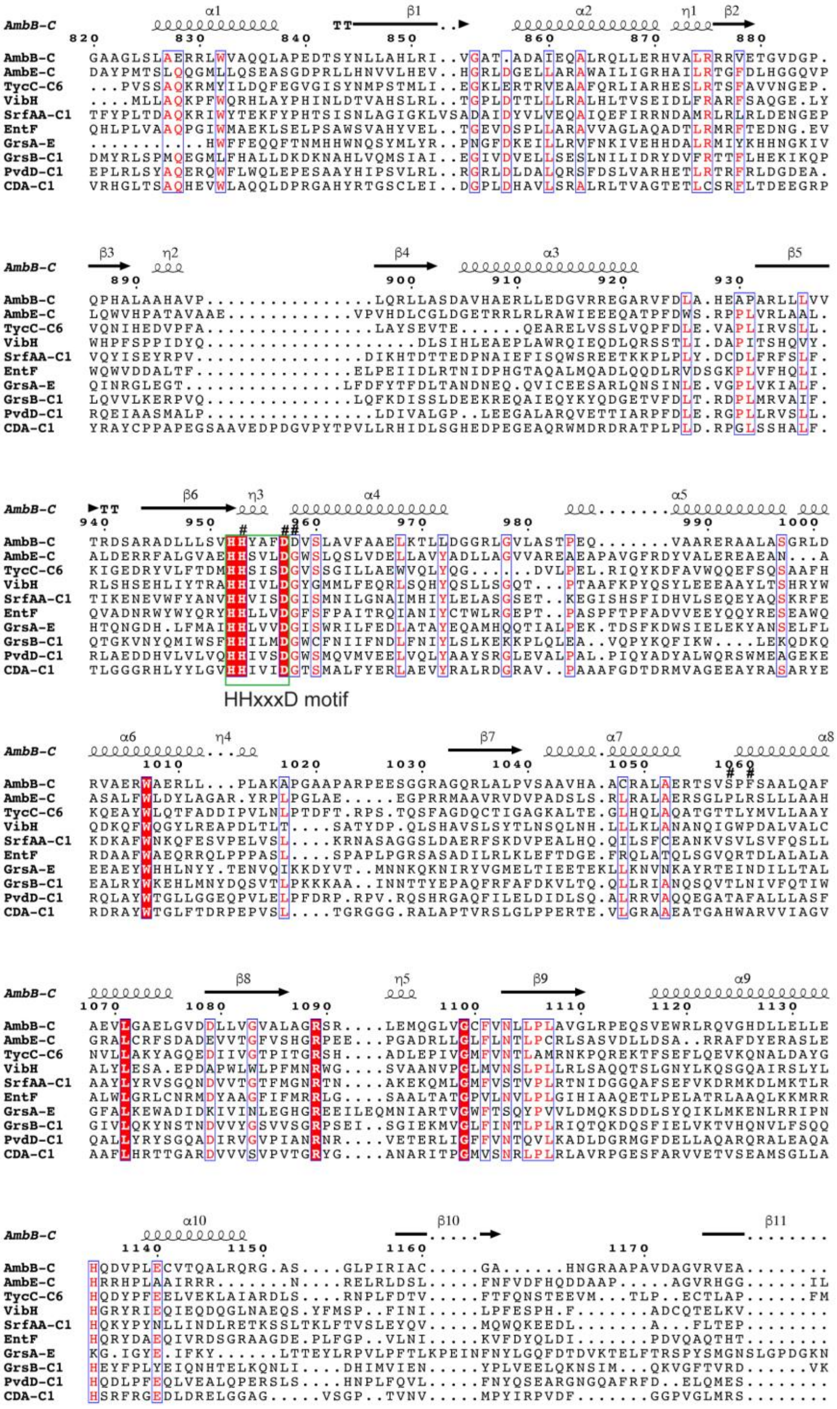

Supplementary Figure. 2 Sequence alignments of C domain of AmbB with homologs from other characterized NRPS. The secondary structures from the C domain of AmbB are shown in the top. The mutated residues are marked with "#". Alignment was created in Clustal Omega [1] and rendered in ESPript 3 [2].

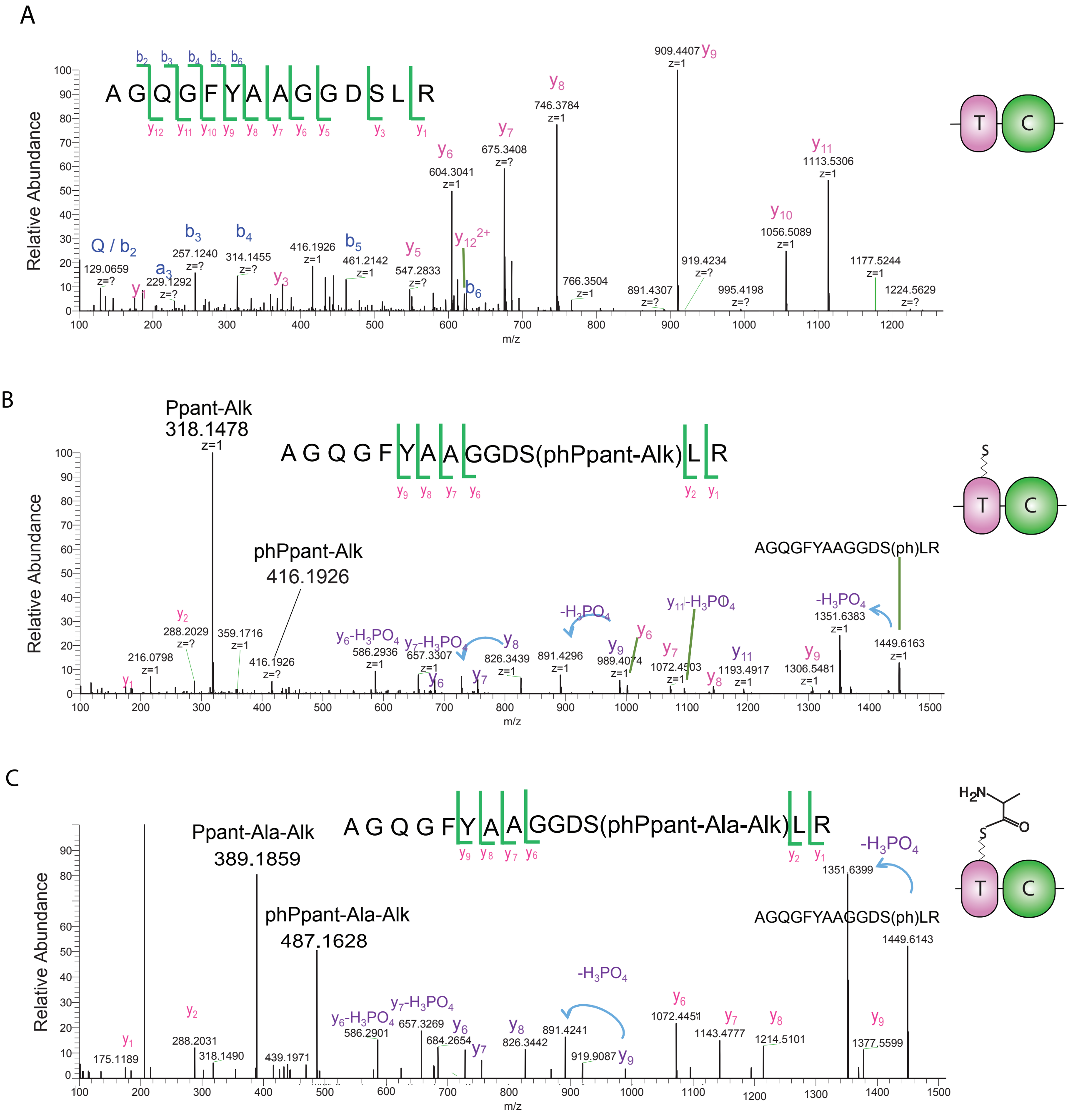

Supplementary Figure 3. LC-MS analysis of apo and holo AmbB T-C. (**a**) Peak profile of the peptide AGQFYAAGGD**S**LR corresponding to amino acids 757-770 of AmbB T-C prior to addition of Ppant. (**b**) Peak profile of the peptide AGQFYAAGGD**S**LR with Ppant-Alkylated (Alk) covalently tethered to the conserved serine of holo AmbB T-C. A peak with m/z 318.14 was observed, which corresponds to Ppant-Alk. (**c**) Peak profile of the peptide AGQFYAAGGD**S**LR showing the presence of a peak with m/z 389.1859, which corresponds to Ppant-L-Ala-Alk.

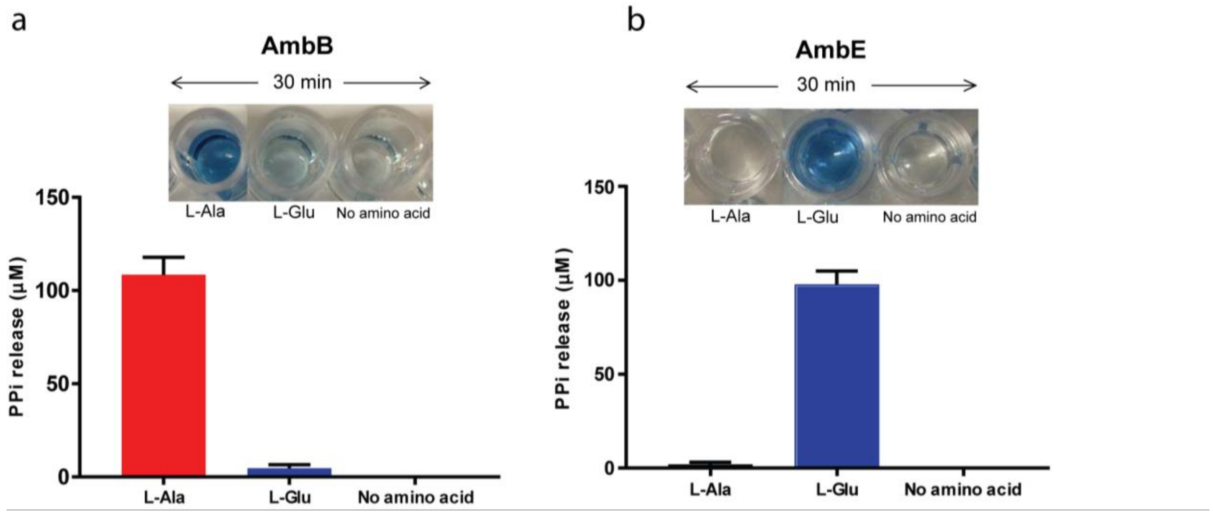

Supplementary Figure. 4 Substrate specificity of AmbB and AmbE. Pyrophosphate (PPi) release detected by the colorimetric assay [3] in a reaction mixture containing 100 µg/mL of either AmbB or AmbE with L-Ala, L-Glu or without amino acid (control) incubated for 30 min, shows that **(a)** A domain of AmbB activates L-Ala while **(b)** A domain of AmbE activates L-Glu. Absorbance was measured at 620 nm in a plate reader. Values represent mean data of three experiments ± SD.

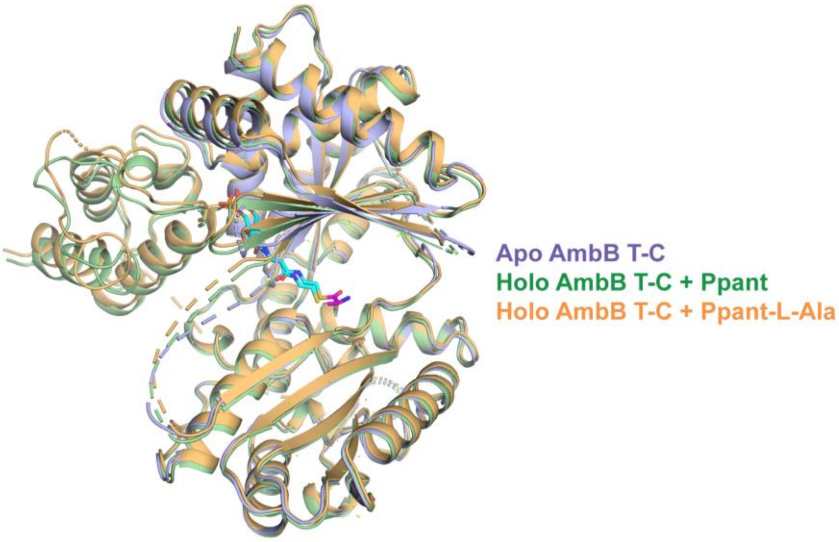

Supplementary Figure. 5 Cartoon representation of superimposed structures of apo and holo AmbB T-C. Superposition of C domain of AmbB T-C with Ppant (green), and AmbB T-C with Ppant-L-Ala (orange) on apo C domain (light purple) show quasi identical fold with RMSD values of 0.54 Å and 0.55 Å, respectively. Superposition of T domain of the two holo AmbB T-C shows RMSD value of 0.26 Å. The T domain is disordered in apo AmbB, and thus not seen in the structure.

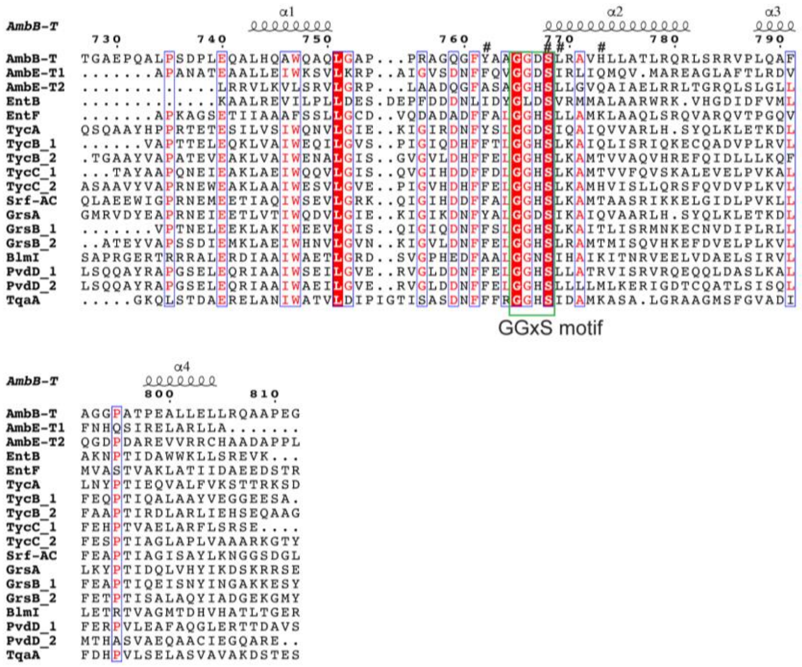

Supplementary Figure. 6 Sequence alignments of T domain of AmbB with homologs from characterized NRPS. The secondary structures from the T domain of holo AmbB T-C are shown in the top. The mutated residues are marked with "#". Alignment was created in Clustal Omega [1] and rendered in ESPript 3 [2].

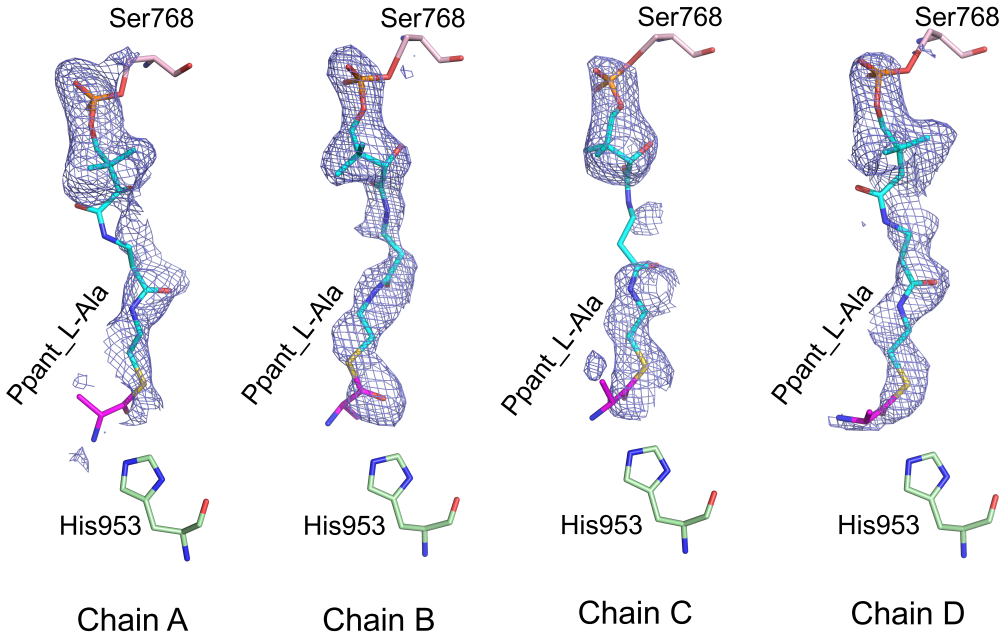

Supplementary Figure 7. Unbiased Fo-Fc map (contoured at 2.5 s) observed prior to building the ligand into the electron density map of holo AmbB-L-Ala. Density for the ligand is satisfactory in two of the four chains (chains B and D) while it is fragmented in the remaining chains (chains A and C).

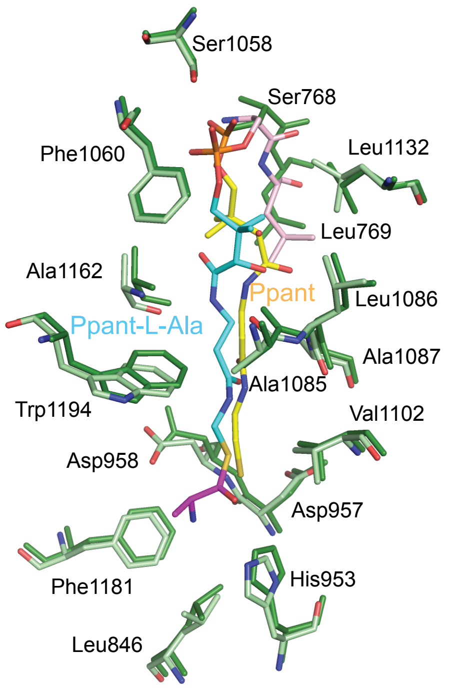

Supplementary Figure. 8 Superimposition of holo AmbB T-C and AmbB T-C tethered with Ppant-L-Ala showing residues surrounding Ppant arm and Ppant-L-Ala, respectively, in donor pocket of C domain of AmbB. Residues of T and C domain of holo AmbB are shown dark green, whereas the residues of T and C domains of holo AmbB with L-Ala are depicted as pink and pale green, respectively.

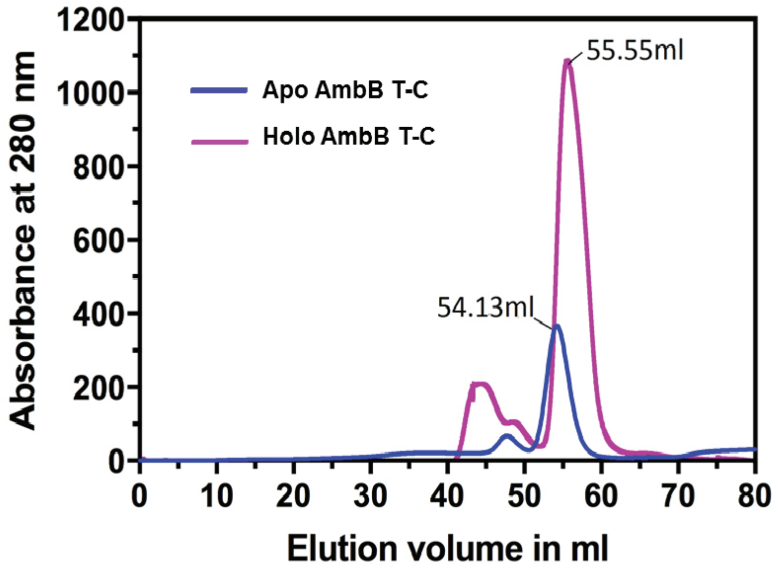

Supplementary Figure. 9 Gel filtration profiles for AmbB T-C. Apo AmbB T-C (blue line) eluted earlier than holo AmbB T-C (pink line).

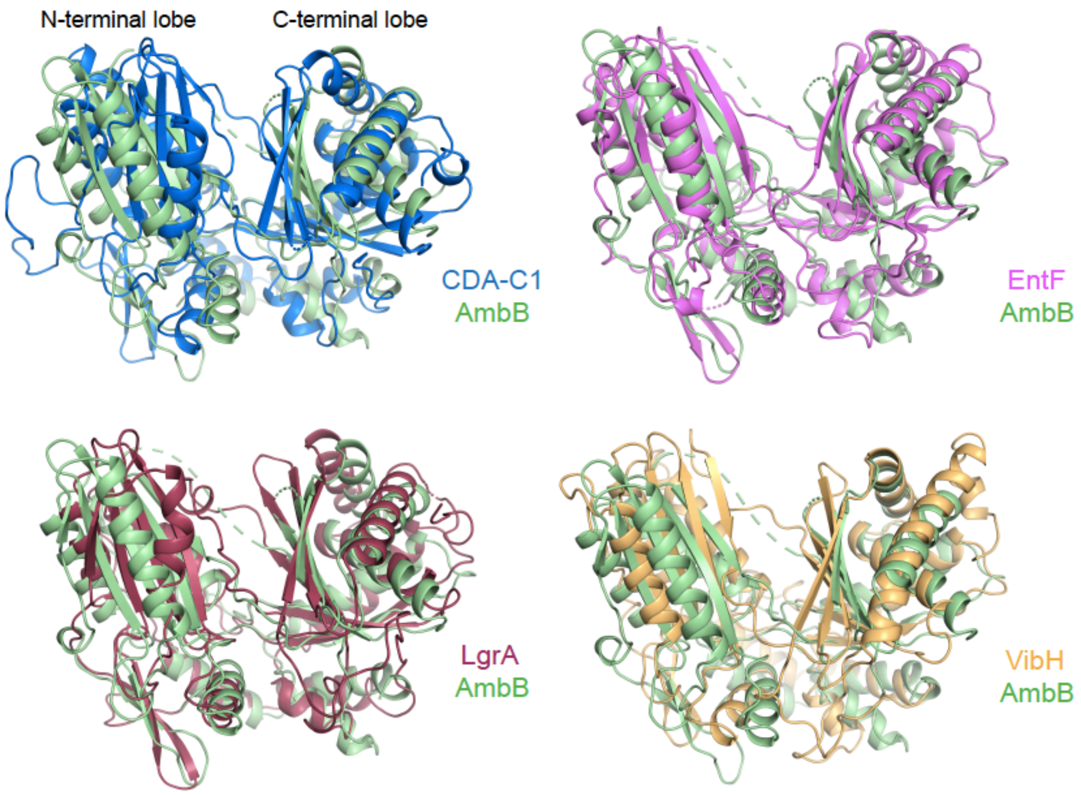

Supplementary Figure. 10 Different conformations of the N- and C-terminal lobes of C domain observed among NPRS. Superimposition of the C-terminal subdomain of AmbB on three characterised C domain structures (VibH, LgrA, EntF) of NRPS, showing varying degree of "openness' of the protein.

Table S1

Data collection and structure refinement statistics

Values in parentheses refer to the highest resolution shell.

	Apo AmbB T-C semet	Apo AmbB T-C	Holo AmbB T-C	Holo AmbB T-C with L-Ala
Space group	P 6_5 2 2	P 6_5 2 2	C 1 2 1	C 1 2 1
Cell parameters a, b, c (Å) α, β, γ (°)	87.88, 87.88, 286.3 90, 90, 120	87.91, 87.91, 285.42 90, 90, 120	198.41, 71.19, 172.77 90, 109.92, 90	198.50, 71.61, 173.76 90, 110.14, 90
Resolution range (Å)	45.0-2.40 (2.53-2.40)	52.06-2.10 (2.21-2.10)	49.56-2.20 (2.25-2.20)	48.88-2.50 (2.56-2.50)
Rmerge	0.35 (3.0)	0.08 (0.65)	0.066 (0.464)	0.067 (0.753)
Total reflections	779682 (83770)	132024 (19638)	291347 (14697)	271707 (15699)
Unique reflections	26538 (3763)	38582 (5494)	114170 (5650)	78940 (4499)
I/σI	12.0 (2.0)	9.8 (2.2)	8.7 (2.1)	11.4 (1.7)
CC 1/2	0.98(0.70)	0.98(0.52)	0.984 (0.507)	0.997 (0.559)
Multiplicity	29.4 (22.3)	3.4 (3.6)	2.6 (2.6)	3.4 (3.5)
Completeness (%)	100(100)	98.8 (98.9)	99.0 (99.0)	99.2 (99.6)
Estimated B factor from Wilson plot (Å^2)	36.7	39.8	26.6	50.7
Rwork		0.20	0.21	0.25
Rfree		0.24	0.24	0.29
RMSD bond length (Å)		0.01	0.009	0.006
RMSD bond angle (°)		1.4	1.3	1.2
Ramachandran plot:				
Favoured region (%)		98.63	97.72	97.7
Additionally allowed region (%)		1.37	2.05	1.76
Outliers (%)		0	0.23	0.54
Average B-factor (Å^2)			**No of atoms /B-factor**	**No of atoms /B-factor**
Protein atoms			13522/44.46	13895/ 60.44
Ligand atoms			84/70.08	78/ 81.951
Solvent molecules			993/44.3	361/ 46.68

Supplementary Table 2.

Primers used in this study. All sequences are in the 5'→ 3' order with restriction enzyme site underlined. Base pair substitution is coloured in red.

Primer	Sequence
**For protein expression**	
AmbB T-C Fwd	cgcggatccaccggcgccgagccgcag
AmbB T-C Rev	ccgctcgagggaagcgttgcagccctccgg
AmbB full-length Fwd	ccggaattcatgcaggagcgacatggc
AmbB full-length Rwv	cccaagctttcaggaagcgttgcagcc
AmbE full-length Fwd	cccaagcttggttgccaggttcgccgg
AmbE full-length Rev	gggaattccatatgagtgcgtcagaagacctgcaatc
**For site-directed mutagenesis**	
AmbB Y762D Fwd	acagggcttcgacgccgccggcggc
AmbB Y762D Rev	ccggcgcgcggcggcgca
AmbB S768AFwd	cggcggcgatgccctgcgggc
AmbB S768A Rev	gcggcgtagaagccctgtccgg
AmbB L769S Fwd	cggcgattcctcgcgggcggtgc
AmbB L769S Rev	ccggcggcgtagaagccc
AmbB H773A Fwd	gcgggcggtggccctgctcgcgac
AmbB H773A Rev	agggaatcgccgccggcg
AmbB H953A Fwd	cagcgtccatgcctacgccttcgacgatgtgtcg
AmbB H953A Rev	agcaggaggtcggcgcgc
AmbB D957A Fwd	tacgccttcgccgatgtgtcg
AmbB D957A Rev	gtgatggacgctgagcag
AmbB D958A Fwd	gccttcgacgctgtgtcgctg
AmbB D958A Rev	gtagtgatggacgctgagc
AmbB S1058A Fwd	acctcggtagccccgttcagtgctgcgctacag
AmbB S1058A Rev	cgttcggccagcgcccgg
AmbB F1060S Fwd	gtaagcccgtccagtgctgcg
AmbB F1060S Rev	cgaggtgcgttcggccag
AmbE S1819A Fwd	cggcgggcatgcgctgctggg
AmbE S1819A Rev	gcgctggcgaagccctgg

References

1. Madeira, F. et al. The EMBL-EBI search and sequence analysis tools APIs in 2019. *Nucleic Acids Res* **47**, W636-W641 (2019).

2. Robert, X. & Gouet, P. Deciphering key features in protein structures with the new ENDscript server. *Nucleic Acids Res* **42**, W320-4 (2014).

3. Maruyama, C., Niikura, H., Takakuwa, M., Katano, H. & Hamano, Y. Colorimetric Detection of the Adenylation Activity in Nonribosomal Peptide Synthetases. *Methods Mol Biol* **1401**, 77-84 (2016).
